# Attitudes of the Portuguese population towards advance directives: an online survey

**DOI:** 10.1186/s12910-024-01043-x

**Published:** 2024-04-03

**Authors:** João Carlos Macedo, Luísa Castro, Rui Nunes

**Affiliations:** 1 Nursing School, University of Minho, Campus de Gualtar, Braga, 4710-057 Portugal; 2 Centre for Health Technology and Services Research (CINTESIS@RISE), Department of Community Medicine, Information and Health Decision Sciences (MEDCIDS), Faculty of Medicine, University of Porto, Porto, 4200-450 Portugal; 3 Center of Bioethics of the Faculty of Medicine, University of Porto, Porto, 4200-450 Portugal

**Keywords:** Advance directives, Attitude, Autonomy, End-of-life, Living will, Durable power of attorney for health care

## Abstract

**Background:**

Advance directives (ADs) were implemented in Portugal in 2012. Although more than a decade has passed since Law 25/2012 came into force, Portuguese people have very low levels of adherence. In this context, this study aimed to identify and analyse the attitudes of people aged 18 or older living in Portugal towards ADs and to determine the relationships between sociodemographic variables (gender/marital status/religion/level of education/residence/whether they were a health professional/whether they had already drawn up a living will) and people’s attitudes towards ADs.

**Methods:**

An online cross-sectional analytical study was conducted using a convenience sample. For this purpose, a request (email) that publicized the link to a –form—which included sociodemographic data and the General Public Attitudes Toward Advance Care Directives (GPATACD) scale—was sent to 28 higher education institutions and 30 senior universities, covering all of mainland Portugal and the islands (Azores and Madeira). The data were collected between January and February 2023.

**Results:**

A total of 950 adults from completed the online form. The lower scores (mean 1 and 2) obtained in most responses by applying the GPATACD scale show that the sample of the Portuguese population has a very positive attitude towards ADs. The data showed that women, agnostics/atheists, health professionals and those who had already made a living will had more positive attitudes (*p* < 0.001) towards ADs. There were no statistically significant differences in the attitudes of the Portuguese population sample towards ADs in relation to marital status, education level, and residence.

**Conclusion:**

The results obtained enable us to confirm that this sample of the Portuguese population has a positive attitude towards ADs. We verify that there are certain fringes of this sample with certain sociodemographic characteristics (women, agnostics/atheists, health professionals and those who had already made a living will) that have a more positive attitude towards ADs. This data could facilitate the implementation and adjustment of relevant measures, particularly in the field of health education and aimed at groups with less favourable attitudes, to increase the effectiveness of voluntary exercise of citizens’ autonomy in end-of-life care planning.

**Supplementary Information:**

The online version contains supplementary material available at 10.1186/s12910-024-01043-x.

## Background

The principle of autonomy has become capitally important in the context of health care [1, 2]. The assumption of the dignity of the human person, which presupposes full respect for the right to self-determination in all areas and particularly in health care, has been developing and taking the place of the paternalistic paradigm that, for a long time, presided over the relationship between users and health care professionals [3–5]. This paradigm shift has accompanied the overwhelming development of technology and knowledge in the medical field that we have witnessed in recent decades and that has allowed us to live longer, even if we have serious illnesses. However, this shift raises many ethical questions: Does the person live with quality of life? Does the person want to live under these circumstances? And when the person is unable to decide, what would their wishes be? In situations of serious illness with no prospect of a cure, therapeutic and diagnostic interventions are often futile [6, 7], leading to practices of prolonging biological life [8, 9] that may not respect the person’s wishes. It seems that the vast majority of people ’do not want this kind of treatment. In this context, in the 1960s in the USA, Attorney Luis Kutner decided, together with his client, who had a serious and incurable illness, to write a document setting out the health care he refused and/or wished to receive if he was unable to express his wishes autonomously. In that way, health care professionals would know in advance what his values and wishes were at the end of his life [10, 11]. This document was disseminated around the world [12, 13], and a consensus was reached in the field of bioethics that it was no longer enough to respect the autonomy of the person with regard to health care in which express informed consent was given. It was also permissible to respect the wishes of people who, for reasons of illness, were now unable to express themselves but who had written down their wishes regarding the health care they wished to refuse and/or receive in end-of-life situations. This form of respect for prospective autonomy, known as advance directives (ADs), has become what some call a breakthrough and a civilizational milestone [14, 15].

In the literature on decision-making about end-of-life care, the terms ADs and advance care planning (ACP) invariably appear. ACP, as defined by consensus, “…is a process that supports adults at any age or stage of health in understanding and sharing their personal values, life goals, and preferences regarding future medical care [16]. ADs “are written documents that specify the medical preferences of competent people” [17]. In pragmatic terms and with the evolution of bioethical reflection, ADs, which are free, nonmandatory instruments that depend on citizens’ choice to comply, are the final producers of planning and can take two different forms that are not mutually exclusive, as both options could be combined [5, 14, 17]:

- Living wills, a document in which the person expresses the health care they refuse or wish to receive if they are unable to express their will autonomously;
- Durable power of attorney for health care, which allows individuals to appoint health care proxies to make health care decisions on their behalf once they lose the ability to do so.

Portugal has also taken this path towards establishing advance directives. In 2001, the Portuguese Parliament ratified the so-called Convention on Human Rights and Biomedicine (1997) of the Council of Europe [18] under Article 9 of the convention, which states:

*“Previously expressed wishes*

*The previously expressed wishes relating to a medical intervention by a patient who is not, at the time of the intervention, in a state to express his or her wishes shall be taken into account.”*

Thus, Portuguese people could put their wishes in writing, and they would be considered by health professionals when they were unable to express themselves autonomously. Despite being a step forwards, this legislation was still not very open. In addition to not being binding on health professionals, it also did not allow people to appoint health care proxies to make health care decisions on their behalf once they lost the ability to do so [5]. 

In 2006, the Portuguese Association of Bioethics launched a challenge to politicians to legalize ADs [19], but it was only later, in 2012, that the Portuguese parliament approved Law 25/2012 [19], which allowed citizens to make an AD, i.e., access to the right to exercise prospective autonomy. In other words, this law provides citizens with the right to create a living will and/or durable power of attorney for health care [20].

Over the years since the law came into force, Portuguese adherence to ADs has been low with approximately 53,000 registrations, corresponding to approximately 0.5% of the Portuguese population [21], which is in line with the findings in other countries, particularly in Europe [22–25]. However, in 2017, the Portuguese Parliament, noting this lack of adherence, asked the government to invest in publicizing this right among the population [26] There has been some publicity on television and social media, but according to the data we have obtained thus far, there has been no significant change in Portuguese people’s adherence to ADs.

It seems that there is a need to study people’s attitudes towards ADs to better understand why this is the situation. Studies in the literature have shown that people mostly have a positive attitude towards ADs [27–30]. Additionally, several studies have shown a correlation between certain sociodemographic data and a positive attitude towards the completion of ADs [27, 31].

However, empirical studies of the Portuguese population are scarce [29, 32], leading us to question the reasons for this situation. In this context, we conducted this study to identify and analyse the attitudes of the population living in Portugal towards ADs and determine the relationships between sociodemographic variables (gender/marital status/religion/level of education/residence/whether they were a health professional/whether they had already drawn up a living will) and people’s attitudes towards ADs.

With regard to predicting the expected results, we propose the following hypotheses:

The Portuguese population has positive attitudes towards ADs;

There is a significant difference in the Portuguese population’s attitudes towards ADs in relation to gender, marital status, religion, education, residence, being a health professional, and having a living will.

## Methods

### Study design and participants

This study describes a cross-sectional analysis of the attitudes of the Portuguese population towards ADs. Data consisted in a convenience sample were collected from January 2023 to February 2023. For data collection, we considered the geographical criterion and a high number of adults to be aggregated and we thought that dissemination to public institutions or social associations with a nationwide presence would be ideal for reaching a more representative sample. Therefore, an email with the Google Forms survey link was sent to 28 higher education institutions (universities and polytechnic institutes) randomly selected but covering all of mainland Portugal and the islands (Azores and Madeira), and they were asked to disseminate the questionnaire to students, teachers, and staff. In addition, to increase the likelihood of receiving a response from the elderly population (aged 65+) with access to the internet, an email was also sent asking 30 senior universities to disseminate the questionnaire. We consider this snowball sampling method to be the most suitable for identifying the largest number of respondents from all over the country.

The minimum sample size was computed based on a proportion calculation, targeting a 95% confidence level. We assumed a worst-case scenario in which the proportion in the population was 0.5 and aimed for a margin of error no greater than 5%. This led to the requirement of a minimum sample size of 384 participants. The reporting of the study followed the guidelines of the Strengthening the Reporting of Observational Studies in Epidemiology (STROBE) [33]. 

### Instruments

The Google Form consisted in three parts: Part I - Information about the research; Part II - Sociodemographic data, which included gender (Male/Female/Other), age (quantitative), marital status (Married/Living together/Divorced/Separated/Single/Widowed), religion (Catholic, Agnostic/Atheist/Other), level of education (Elementary education/Secondary education/Bachelor’s degree/Master’s degree/Doctorate), district of residence (all districts of Portugal available for selection), whether they were a health professional (yes/no) and whether they had already drawn up a living will (yes/no); and Part III - General Public Attitudes Toward Advance Care Directives Scale (GPATACD).

The researchers requested authorization to use the General Public Attitudes Toward Advance Care Directives Scale (GPATACD) from the authors who validated it for the Portuguese population [34]. The authors of the scale’s validation obtained a Cronbach’s alpha of 0.848, indicating adequate internal consistency.

The GPATACD instrument has 26 statements answered on a 5-point Likert scale (completely disagree = 1 and completely agree = 5). The scores range from 1 to 5, and lower scores reflect more positive attitudes. The scale has 4 factors and their respective dimensions are: F1 – a person’s autonomy and dignity at the end of life (includes statements no. 1,2,8,9,11 15 and 18); F2 - decision-making at the end of life (statements no. 6,7,10,12); F3 - decision-making at the end of life (groups together statements no. 6,7,10,12,13,16,17 and 19); F3 - application of ADs (statements 3,4,5,14,20 and 23); and F4 - perceptions about the end of life (statements 21,22,24 25 and 26) [34].

In the present study, a Cronbach’s alpha of 0.790 was obtained for the total scale, indicating adequate internal consistency.

Before we began collecting the data, we pretested the GPATACD scale with a group of randomized participants (aged 18 or older) who were sent an email with a link to the form (*n* = 30) to check that the language was appropriate and that each item on the scale was easy to understand. Some of the participants had doubts about the interpretability of some of the scale’s items. We made minor changes to the wording of Items 17, 18 and 19. Item 17 was originally described as follows: “My family will make end-of-life decisions for me, when necessary” was changed to “My family will make end-of-life decisions for me when I am unable to do so”. Item 18, which was originally described as “I will overwhelm my family with end-of-life decisions”, was changed to “I will overwhelm my family with end-of-life decisions if I am unable to make decisions autonomously”. Item 19, which was originally described as “My doctor will make end-of-life decisions for me when the time comes”, was now worded as follows: “The medical team will make the end-of-life decisions if I am unable to do so”. The consensus for this amendment was reached after consultation with two language experts and validation by the team of researchers.

We conducted an additional test with another 30 randomized participants, and this time, there was no doubt about the interpretability of the scale.

### Ethical procedures

The study was approved by the Ethics Committee of the Faculty of Medicine of the University of Porto (protocol approval n.° 59/CEFMUP/2022). All study procedures followed the recommendations of the Declaration of Helsinki and subsequent updates. All participants were assured of the anonymity and confidentiality of all the data provided. The participants were informed in advance about the aims of the study and had to consent to voluntary participation to continue completing the form. There was no monetary or other payment for taking part in the study.

### Data analysis

The data were analysed using IBM SPSS (Statistical Package for the Social Sciences, IBM, version 28.0). We used the tool “detection of duplicates”, which is available in SPSS, to catch duplicate data, but no duplicates were found. Categorical variables were characterized using absolute and relative frequencies, n (%). The normality of the quantitative variables was assessed by examining the histogram. Since most of the quantitative variables exhibited non-symmetric distributions, they were all summarized using the median and corresponding interquartile interval (1st Q; 3rd Q). The items of the scale, which were ordinal in nature, were also described by medians and interquartile intervals.

The internal reliability of the GPATACD scale was evaluated using Cronbach’s alpha (α), and a value exceeding 0.7 was considered satisfactory [35].

Multiple linear regression analysis was conducted for the GPATACD scale as the outcome. The selection of independent variables was based on initial simple linear regressions involving the following variables of interest: gender, age, religion, level of education, whether one was a health professional and finally whether the participant had already drawn up his or her living will. All variables that exhibited correlations with the outcomes at a significance level of *p* ≤ 0.2 in the simple regression analyses were included in the subsequent multiple linear regression analysis. Ultimately, only the variables that demonstrated statistical significance (*p* ≤ 0.05) were retained in the final multivariate model.

The results of the linear regression analysis are presented as nonstandardized coefficient values (β), 95% confidence intervals (95% CIs), and *p* values. The model’s performance was assessed by considering the F-statistic from the overall model test, *p* values, and coefficients of determination (R²). The final linear regression model was validated through the following steps: (a) visual assessment of the residuals’ normality via histograms; (b) application of a t test to ascertain whether the mean residuals equalled zero; and (c) generation of plots depicting residuals against fitted predictive values to assess homoscedasticity. p values ≤ 0.05 indicated statistical significance.

## Results

### Sociodemographic data

In total, 950 complete responses were obtained for this e-survey (with the exception of 5 nonresponses to the item on sociodemographic characteristics related to religion; see Table 1); therefore, there was no processing of missing data. This convenience sample revealed that the age of the participants ranged from 18 to 83 years, with a median of 45 years (Q1-34 and Q3-54). The majority of participants were women (*n* = 622; 65.5%) and were married or in a de facto union (*n* = 547; 57.6%). In religious terms, the majority said they were Catholic (*n* = 686; 72.6%). In terms of educational qualifications, the majority had a higher education (bachelor’s degree, *n* = 402 − 42.3%; master’s degree, *n* = 233 − 24.5%; doctorate = 139 − 14.6%). In demographic terms, since we used the European statistical nomenclature NUTS II - Nomenclature of Territorial Units for Statistics [36], we found that the majority of the respondents were from the northern region of Portugal (*n* = 598; 62.9%), although, as aforementioned, we received responses from all regions of the country. The majority of the respondents were not health professionals (*n* = 624; 65.4%). Finally, the majority of respondents had not drawn up a living will (*n* = 916; 96.4%) (Table 1).

**Table 1:** –Sample sociodemographic characteristics (*n* = 950)

Variable	Descriptive
**Age : median (1st °Q;3rd °Q)**	45 (34;54)
	**n (%)**
**Gender**	
Mal	324 (34.1)
Female	622 (65.5)
Other	4 (0.4)
**Marital status**	
Married/living together	547 (57.5)
Divorced/separated	89 (9.4)
Single	303 (31.9)
Widowed	11 (1.2)
Religion *	
Catholic	686 (72.6)
Agnostic/Atheist	231 (24.4)
Others	28 (3)
**Level of education**	
Elementary education	18 (1.9)
Secondary education	158 (16.6)
Bachelor´s degree	402 (42.3)
Master´s degree	233 (24.5)
Doctorate	139 (14.7)
**Residence (NUTS II)**	
North	598 (63)
Algarve	4 (0.4)
Center	194 (20.4)
Lisbon Metropolitan Area	93 (9.8)
Alentejo	24 (2.5)
Autonomous Region of the Azores	23 (2.4)
Autonomous Region of the Madeira	14 (1.5)
**Are you a health professional?**	
No	626 (65.9)
Yes	324 (34.1)
**Have you prepared your living will?**	
No	916 (96.4)
Yes	34 (3.6)

* 5 participants did not answer

### Attitudes towards Advance directives

The results obtained by applying the GPATACD scale, in which lower scores reveal a more positive attitude, suggest a positive attitude towards Advance Directives (Table 2). We found responses with a median of 1 in 11 statements (no. 1,2,4,6,8,10,11,12,14,15 and 20) and with a median of 2 in 7 statements (no. 3,5,7,9,13,16 and 26). These lower values in the answers to the scale show that the majority of the respondents had a very positive attitude. Regarding the highest values, a less favourable attitude towards ADs, we obtained a median of 3 out of 4 statements (no. 17, 19, 23 and 25) and a median of 4 out of 3 statements (no. 18, 21, 22 and 24).

With regard to the factors and respective dimensions of the scale, which we mentioned in point 2.2, the total score was low (median = 2.27; Q1-2.04; Q3-2.54), indicating a favourable attitude towards ADs (Table 2). However, the highest score was found for F4-Perception of the EOL, with a median of 3.4 (Q1-2.04; Q3-3.8), indicating less favourable attitudes in this area.

**Table 2:** Descriptive statistics of the GPATACD scale (n= 950)

N.°/Items	Median (1^st^ Q 3^rd^ Q, min-max
1 – The existence of the vital testament is not important.	1 (1;2), 1–5
2 – My opinion should not be respected in the end-of-life process.	1 (1;1), 1–5
3 - Advance directives do not reflect the patient’s values and preferences when making therapeutic decisions at the end-of-life.	2 (1;2), 1–5
4 –Advance directives are a useful tool for health care professionals when making decisions about end-of-life patients.	1 (1;2), 1–5
5 - The health care prosecutor appointed by the patient does not facilitate the professionals’ decision-making.	2 (1;3), 1–5
6 – Compliance with advance directives concerns the physician.	1 (1;2), 1–5
7 – Advance directives are a legal form of euthanasia.	2 (1;3), 1–5
8 – It is not important that patients make their vital testament or advance directives.	1 (1;2), 1–5
9 - It is not important that all citizens make their vital testament or advance directives.	2 (1;2), 1–5
10 – Advance directives are important only for religious reasons.	1 (1;2), 1–5
11 – Legalization of the vital testament did not contribute to human dignity.	1 (1;2), 1–5
12 - Death must be postponed, regardless of the person’s condition.	1 (1;2), 1–5
13 – End-of-life care should be provided based on the opinion of the health professional.	2 (2;4), 1–5
14 – End-of-life care should not be provided based on the patient’s opinion.	1 (1;2), 1–5
15 – I do not want to be able to have an opinion on the care I can receive in an end-of-life situation	1 (1;2), 1–5
16 - End-of-life care should be provided based on the opinion of the family.	2 (1;3), 1–5
17 - My family will make end-of-life decisions for me when I am unable to do so.	3 (2;4), 1–5
18 - I will overwhelm my family with end-of-life decisions if I am unable to make decisions autonomously.	4 (3;4), 1–5
19 -The medical team will make the end-of-life decisions if I am unable to do so.	3 (2;4), 1–5
20 – The vital testament is only important for elderly and sick people.	1 (1;2), 1–5
21 – I am currently healthy, but there may be a need to consider decisions regarding the final phase of my life.	4 (4;5), 1–5
22 – At my current age, there may be a need to consider end-of-life decisions.	4 (3;5), 1–5
23 – I have information on Advance directives/vital testament.	3 (2;4), 1–5
24 – It is possible to make end-of-life decisions, even if I cannot imagine myself in such a situation.	4 (3;5), 1–5
25 – I do not make vital testament because there is still little information available.	3 (2;3), 1–5
26 – I do not want to think that I will eventually die or become disabled to the point of not being able to make decisions.	2 (2;4), 1–5

As there is evidence that sociodemographic factors can influence people’s attitudes towards ADs [31, 37–41], multiple linear regression analyses were conducted (Table 3). The multiple models obtained reasonably comply with the assumptions of normality of the zero-mean residuals, homogeneity of variance, and VIF between 1.01 and 1.02 (tolerance 0.981–0.991). The results showed, after we adjusted for the remaining variables in the model, that women scored, on average, 0.14 lower than men (*p* < 0.001) on the GPATACD Scale; agnostics/atheists scored, on average, 0.11 lower than Catholics; health professionals scored, on average, 0.14 lower than nonhealth professionals; and finally, those who had drawn up their living will scored, on average, 0.19 lower on the scale than those who do not have a living will. No statistically significant results were obtained for the variables marital status, education, or residence when adjusting for the remaining variables.

**Table 3:** Descriptive statistics of the GPATACD scale and its dimensions (*n* = 950)

Scale and subscales	Median (1st Q; 3rd Q)	Min–Max	Cronbach`s alpha
F1 – Autonomy and dignity of the person at the EOL	1.71 (1.57; 2.14)	1-4.43	0.681
F2 - EOL decision-making	2.13 (1.75; 2.63)	1-3.88	0.702
F3 - Application of ACD	2 (1.67; 2.33)	1-4.5	0.470
F4 - Perception of the EOL	3.4 (3.2; 3.8)	1–5	0.438
GPATACD Total Score	2.27 (2.04; 2.54)	1-3.77	0.790

## Discussion

This is the first nationwide study in Portugal to evaluate attitudes towards ADs. We received responses from all administrative areas of the country (see Table 1), although there was a predominance of responses from the northern part of the country. The results obtained from this sample showed a favourable attitude towards ADs (Table 2), confirming our first hypothesis, which is in line with the findings of other studies and in different countries [23, 28, 30, 31, 41–45]. Notably, the data obtained from this sample revealed a more positive attitude among women, agnostics/atheists, health professionals, and those who had already performed an AD (Table 4). This result confirms our hypothesis that there was a statistically significant difference in the attitudes of the Portuguese population towards ADs in relation to gender, religion, being a health professional and having a living will. On the other hand, we did not confirm the other hypotheses, as we did not find a statistically significant difference in the attitudes of the Portuguese population in relation marital status, education level and residence.

**Table 4:** Initial and final multiple linear regression models for the GPATACD scale as the outcome (*n* = 950

	Initial Model R^2^ = 9.02%; F(8,932) = 11.6; *p* < 0.001		Final Model R^2^ = 8.47%; F(5,935) = 17.3; *p* < 0.001	
	**B [95% IC]**	**p value**	**B [95% IC]**	**p value**
**Gender**				
*Male*	Reference		Reference	
*Female*	-0.14 [-0.20; -0.09]	**< 0.001**	-0.14 [-0.19; -0.09]	**< 0.001**
**Religion**				
*Catholic*	Reference		Reference	
*Agnostic/Atheist*	-0.11 [-0.16; -0.05]	**< 0.001**	-0.11 [-0.16; -0.05]	**< 0.001**
*Others*	0.09 [-0.06; 0.23]	0.246	0.08 [-0.07; 0.23]	0.285
**Level of education**				
*Bachelor´s degree*	Reference			
*Elementary or secondary education*	-0.07 [-0.14; 0.002]	0.056		
*Master´s degree*	-0.06 [-0.12; 0.006]	0.074		
*Doctorate*	-0.01 [-0.09; 0.06]	0.793		
**Are you a health professional?**				
*No*	Reference		Reference	
*Yes*	-0.15 [-0.21; -0.09]	**< 0.001**	-0.14 [-0.19; -0.09]	**< 0.001**
**Have you prepared your living will?**				
*No*	Reference		Reference	
*Yes*	-0.22 [-0.36; -0.09]	**0.001**	-0.19 [-0.36; -0.008]	**0.001**

Abbreviations – B: nonstandardized regression coefficient; CI: confidence interval; R²: coefficient of determination

Another study conducted by Laranjeira et al., that was also with Portuguese citizens showed results that resembled those of the present study in terms of the GPATACD scale [29]. However, there are differences between this other study and our study that may explain some of the differences in the results. Thus, the sample in the study by Laranjeira et al. [29] included health care professionals (*n* = 728; 71.09%), whereas in this study, we had only 324 health care professionals, corresponding to 34.1% of the sample. Finally, we had a sample with national coverage.

A similar finding, although slightly greater in our sample, is the number of people who have written an AD. In our study, 3.6% of the participants had already written an AD; in the study by Laranjeira et al. (2021), this percentage was 2.34% of the sample; and in another study, which was also conducted in Portugal in 2017, it was approximately 1.3% [32]. These different, but low, figures are nevertheless higher than the national statistics obtained from the National Living Will Registry in the Portuguese population [46]. In fact, adherence to ADs among the country’s resident population was approximately 0.28% in 2016 and almost doubled to 0.51% in 2022 [47]. In absolute terms, in January 2023, the number of active living wills exceeded 37,000, nearly 13,000 of which were registered by men and more than 24,000 by women [21].

Curiously, this national data, in which 64% of the population who have drawn up a living will are women, are in line with the data from this study, which shows that women have a more favourable attitude towards living wills than men, i.e., they are more likely to discuss the subject and more easily draw up their living wills, in line with other empirical data [31, 37, 38, 48–50] Regarding the low level of adherence to developing ADs in the Portuguese population, this is something that is common in almost all countries. With the exception of the USA, where data show that one-third of the population has an AD [51, 52], in Europe, adherence rates vary between 0.66% and 19% [22–25, 29, 53, 54]; Portugal’s percentage is actually lower than the European average.

Although the results obtained show a sample that views Ads favourably, further reflection on some scores obtained in some of the answers on the scale is needed because they have higher score values. Indeed, we can see that some statements obtained median scores of 3 (statements 17, 19, 23, and 25) and 4 (statements 18, 21, 22, and 24), which are higher than those in the study by Laranjeira et al. [29]. These results, with scores that show a less favourable attitude towards ADs, can be partly understood by the composition of this sample, which is mostly composed of nonhealthcare professionals (unlike the study by Laranjeira et al.) and thus contact end-of-life decision-making at the end of life; therefore, perceptions about the end of life are experiences that ordinary citizens have less often than health professionals who start talking about end-of-life issues as early as their initial training, responding with other knowledge on the subject. The results obtained in this study, in which health professionals have a more positive attitude towards ADs, are also understandable and are in line with the findings of some empirical studies [29, 39, 55].

There are likely other barriers that could contribute to these scores showing less favourable attitudes towards ADs. For example, the absence of or little discussion of death and dying in the community [45, 56–59], illiteracy about ADs [23–25, 27, 28, 53, 60–63] and the taboo of death itself [44, 45, 64] may have influenced these results, with these responses showing a less favourable attitude towards ADs [65–67]. As we can see from the studies that have established a positive correlation between the variable’s knowledge about ADs and attitudes [29, 30], we may also see this correlation in these answers: low level of knowledge and less positive attitudes. Notably, 4 of the 8 statements with the highest scores, namely, statements 21, 22, 24, and 25, belong to the dimension of end-of-life perceptions. In this context, there is also illiteracy about end-of-life care, so people’s perception of the subject is more distant, and they do not think about the need to prepare for the end-of-life in terms of health care [45]. On the other hand, the majority of the sample was Catholic (72.6%), which may indicate a tendency to leave the end-of-life to God and not interfere in this matter [68]. Although the Catholic Church has no objection to ADs [69], it is true that this issue is little developed in its official documents. There are notes condemning euthanasia and assisted suicide, but there is no comment about ADs. Furthermore, in a doctrinal line of thought, believers may be faced with the idea of not interfering in God’s designs and opting for a “natural death” (a term often used by the Catholic hierarchy) and meeting the criterion of the sacredness of life, which is more in line with doctrinal precepts. On the other hand, there may also be a gap in communication with the faithful, a lack of knowledge about the church’s position on this issue, which will lead to a position less favourable to ADs. However, the results obtained in this study indicate that agnostics/atheists have a more favourable attitude towards ADs, which is in line with the findings of some studies that point in the opposite direction, i.e., a more religious population is less likely to think about or carry out an AD [70–72].

There is also the likelihood that these results are, like the results of other studies, namely, those in Spain [73], connected to the fact that in the Portuguese population, a certain medical paternalism is still present and still fails to encompass the exercise of autonomy in its fullness as far as health care is concerned, let alone as far as prospective autonomy is concerned. The higher scores in statements 17 and 19, which are part of the end-of-life decision-making dimension, can also be understood considering the lack of or little experience in planning end-of-life care and the lack of dialogue with the family or medical team about end-of-life [30, 39, 63, 74–76]. The results of statement 18 (autonomy and dignity of the person at the end of life) and statement 23 (application of ADLs) should also be mentioned, as higher scores could indicate a lack of knowledge among the Portuguese population about ADs, an aspect that has also been observed in other studies [29, 32, 74].

This study has several implications for education, research and care practice. As we have identified the attitudes of the population towards ADs, we necessarily have, although with the limitations imposed by this study, some suggestions that answer some of the questions we had in this research and launch more ideas to deepen the knowledge and development of ADs in Portugal.

This study could serve to enable politicians and even health professionals to implement and adapt measures to increase the effectiveness of citizens’ autonomy in end-of-life care planning, such as setting up education sessions on ADs [29] and consultations on drawing up a living will [67]. On the other hand, to have a population with a more conscious attitude towards end-of-life care planning, it may be important introduce end-of-life issues into the content of health education [77–80], especially to groups that the evidence shows have less favourable attitudes. We also thought it important to follow the suggestion of the Portuguese Bioethics Association to make it binding for health institutions to inform citizens of their right to draw up an AD [81]. In this context, it will also be important to focus on training health professionals, even though we know from the study that they have a more positive attitude than the rest of the population has, so that they can help citizens with counselling for ADs. Moreover, end-of-life care planning should be introduced into the curricula of health professionals to help shape a more conscious attitude.

This study also revealed the need for further research on ADs in Portugal on an ongoing basis, with representative samples, to obtain additional data on attitudes, the influence of sociodemographic data, and the level of knowledge, barriers and constraints in the compilation of ADs. Only in this way will we be able to provide more appropriate responses to the needs of the population with regard to the right to exercise end-of-life care planning.

### Strengths and limitations

To our knowledge, the major strength of this research is that it is the first study to evaluate attitudes towards ADs with nationwide coverage. However, it has several limitations. First. given that the data collection was conducted online, we were unable to reach 11.8% of Portuguese households, as reported by the National Statistics Institute in 2022, who lacked internet access [82]. On the other hand, the sample may not be representative of the Portuguese population, as we adopted a convenience sample for this research, and the results cannot be generalized. However, they can provide valuable evidence for further research.

As in other studies that utilize e-surveys with voluntary participation, our data may be subject to self-selection bias. This means that individuals who have a greater interest in the topic are more inclined to respond to the questionnaire, potentially skewing the results. Given that dependent and independent variables were measured using the same method (survey) results may also be affected by the common method bias. In particular, participants can respond providing socially desirable responses, potentially inflating the outcomes. The questionnaire was made anonymous mitigating this type of bias.

In addition, we used quantitative methods that allowed us to obtain data on public attitudes towards ADs. Although the data are relevant, there is a limitation in terms of the underlying basis of the attitudes. Therefore, an in-depth qualitative or mixed methods approach exploring Portuguese people’s experiences of AD would be beneficial for future research.

### Conclusion

This study highlights that the attitudes of a sample of the Portuguese population towards ADs are very positive. On the other hand, it also showed that women, atheists/agnostics, health professionals and those who have already drawn up a living will have a more favourable attitude towards living wills.

This research, despite its limitations, could serve to guide policymakers and health professionals in implementing and adapting strategies to make the exercise of citizens’ autonomy in end-of-life care planning more effective. However, it is necessary to continue research on the attitudes of the Portuguese population with representative samples to increase the level of evidence in this field. Thus, we can conclude that we still need to implement measures in education, care practice and research to increase the range of knowledge and awareness of ADs in Portugal.

### Electronic supplementary material

Below is the link to the electronic supplementary material.

Supplementary Material 1

## Data Availability

The General Public Attitudes towards Advance Care Directives (GPATACD) Scale is available for consultation (Additional file 1), and all the data generated or analysed during the study are included in this article.

## Abbreviations

ACP: Advance care planning
ADs: Advance directives
GPATACD: General Public Attitudes toward Advance Care Directives
