# Supplementary material for: Attitudes of the Portuguese population towards advance directives: an online survey
Source: BMC Med Ethics. 2024 Apr 3;25:40. doi: 10.1186/s12910-024-01043-x (PMC10988855; doi:10.1186/s12910-024-01043-x)
Supplement: Supplementary file 1 — Supplementary Material 1 [file 12910_2024_1043_MOESM1_ESM.pdf]

General Public Attitudes Toward Advance Care Directives Scale (GPATACD)

Statements	Completely disagree	Disagree	Neither agree nor disagree	Agree	Completely agree
1 – The existence of the vital testament is not important.					
2 – My opinion should not be respected in the end-of-life process.					
3 - The advance directives do not reflect the patient's values and preferences when making therapeutic decisions at the end-of-life					
4 –Advance directives are a useful tool for healthcare professionals when making decisions about end-of-life patients.					
5 - The health care prosecutor appointed by the patient does not facilitate the professionals' decision-making.					
6 – Compliance with advance directives concerns the physician.					
7 – Advance directives are a legal form of euthanasia.					
8 – It is not important that patients make their vital testament or Advance directives.<br>9 - It is not important that all citizens make their vital testament or advance directives.					
10 – Advance directives are important only for religious reasons.					
11 – Legalization of the vital testament did not contribute to human dignity.					

12 - Death must be postponed, regardless of the person’s condition.					
13 – End-of-life care should be provided based on the opinion of the health professional.					
14 – End-of-life care should not be provided based on the patient’s opinion.					
15 – I do not want to be able to have an opinion on the care I can receive in an end-of-life situation					
16 - End-of-life care should be provided based on the opinion of the family.					
17 - My family will make end-of-life decisions for me when I am unable to do so.					
18 - I will overwhelm my family with end-of-life decisions if I am unable to make decisions autonomously.					
19 -The medical team will make the end-of-life decisions if I am unable to do so.					
20 – The vital testament is only important for elderly and sick people.					
21 – I am currently healthy, but there may be a need to consider decisions regarding the final phase of my life.					
22 – At my current age, there may be a need to consider end-of-life decisions.					
23 – I have information on Advance directives/vital testament.					
24 – It is possible to make end-of-life decisions, even if I cannot imagine myself in such a situation.					
25 – I do not make vital testament because there is still little information available.					
26 – I do not want to think that I will eventually die or become disabled, to the point of not being able to make decisions.					
